# Infrared Thermography Sensor in the Analysis of Acute Metabolic Stress Response during Race Walking Competition

**DOI:** 10.3390/bios14100478

**Published:** 2024-10-05

**Authors:** Alessio Cabizosu, Cristian Marín-Pagan, Pedro E. Alcaraz, Francisco Javier Martínez-Noguera

**Affiliations:** 1 THERMHESC Group, Ribera Hospital de Molina San Antonio, Catholic University of Murcia (UCAM), 30830 Murcia, Spain; 2 Research Center for High Performance Sport, Catholic University of Murcia (UCAM), 30830 Murcia, Spain; cmarin@ucam.edu (C.M.-P.); palcaraz@ucam.edu (P.E.A.); fjmartinez3@ucam.edu (F.J.M.-N.)

**Keywords:** thermography, walkers, sport, exercise tolerance

## Abstract

**Introduction:** Due to the possible impact of the thermoregulatory process on sports performance, it is necessary to explore the existing relationships between kinetic, mechanical, and physiological variables. The objective of this study was to evaluate metabolic stress using thermography in the lower limb after the Spanish Championship 2023 walk. **Method:** A descriptive study was carried out on national and international race walkers before and after the 2023 Spanish Championships. The participants performed different tests within the same circuit. Five walkers completed the long-distance race of 35 km, four walkers completed the middle-distance race of 20 km and finally, two walkers completed the short-distance race of 10 km. **Result:** Statistically significant changes were observed in the lower limbs of the walkers after completing the test. We observed a decrease in skin temperature in all the anatomical regions analyzed, except for the back of the leg. More specifically, the decrease was in the hip (−1.92 °C: *p* = 0.004), quadriceps, hamstrings (−1.23 °C: *p* = 0.002), and tibia (−1.23 °C: *p* = 0.030). However, in the posterior region of the leg, a significant increase in temperature was observed (+0.50 °C: *p* = 0.011) following the competition. **Discussion and Conclusions:** The findings in this descriptive investigation support the notion that thermography may serve as a useful tool in the acute analysis of muscle functional activation and metabolic response in professional marching athletes. Moreover, the results confirmed that the change in skin temperature is the result of a variation in acute metabolic and functional activation in the lower extremities of race walkers during competition, with infrared thermography representing an instrument capable of detecting such a change in a rapid and non-invasive manner.

## 1. Introduction

The International Athletics Federation (IAAF), which is dedicated to the correct development of race walking practices, requires that, with respect to the sagittal axis, “the leg that is placed in front must be in knee extension from the moment it contacts the ground until the moment it reaches full verticality and that the athlete does not lose contact with the ground in a way visible to the human eye” [1]. Given the specific motor coordination required to meet the biomechanical demands of this Olympic specialty, along with the extended competition durations that athletes face, predicting and controlling both articular and muscular metabolic responses is essential for planning energy expenditure, optimizing performance, and ultimately achieving better sports results [2,3].

In fact, in the field of sports medicine, the study of muscle fatigue has significantly expanded in popularity over the last decade, and increasingly, it seems that thermoregulation is a critical element to consider when studying sports performance [4,5], injury prevention, and athlete monitoring systems. This is because heightened skin temperature has been associated with fluid loss, cardiovascular response, and muscle fatigue across several studies [6,7,8].

Biomechanical analysis in relation to thermoregulatory response mechanisms has also gained more research attention in numerous studies more recently [9,10]. Particularly, there is a growing interest in endurance sports such as walking and running [11,12] in order to improve both team and solo performance, advancing the idea that optimization of thermoregulatory response may be key to fatigue management, thus optimizing the relationship between the duration, intensity, and precision of the biomechanical gesture in athletic training in order to obtain better sporting results [13,14].

A recent study published on the world walking champions of Oman 2022 [11] has shown that at different environmental temperatures, the thermoregulation of the walkers varies depending on the anatomical region, showing that the metabolic and blood flow changes were different depending on the anatomical area involved, and this could be due just to the peculiar biomechanics of walking that this type of athletes must perform. However, these findings need to be further elaborated and expanded as they were obtained in a closed laboratory with a relatively small sample size and with incremental tests, while the official tests were conducted in field settings adopting a more linear development in terms of fatigue and metabolic stress. Physiologically, it is known that as molecular agitation increases at the tissue level due to progressively increasing fatigue, changes in infrared radiation emission can be observed at the skin level, so thermography may be showing deeper tissue responses at the surface level in the lower limbs following metabolic stress, which should be tested under different environmental conditions and competition surfaces (dirt, asphalt, treadmill, etc.) [15,16].

However, these authors emphasized the anterior and posterior aspects of the lower extremities, neglecting the role of the hip, despite other studies identifying the hip as a key joint in the development of professional race walkers [17]. In fact, several studies already describe the kinematic characteristics of gait, highlighting the position and activity of the hip and ankle as crucial elements in analyzing and evaluating stride length to enhance athletic performance [18]. Nevertheless, no studies to date have examined these variables using clinical thermography. Due to the possible impact of metabolic stress on sporting performance, and having access to the easy-to-use technology of thermography, it would be of great interest to assess lower limb joints with clinical thermography to accurately determine metabolic changes after long-term competitions.

Therefore, the main objective of this study was to evaluate the acute metabolic stress response by thermography in the lower limbs before and after the Spanish Championships 2023 in race walking. The novelty of this study is due, on the one hand, to the anatomical regions analyzed, and on the other hand, to the type of event and the profile of the participating athletes, as it is the first study to analyze the thermoregulation pattern of the hip joint in an official national event involving world-class and Olympic race walkers. Our hypothesis is that after the competition, there is a decrease in thigh and hip skin temperature.

## 2. Methods

### 2.1. Study Design

A descriptive study was carried out on national and international race walkers before and after the 2023 Spanish Championships. The participants performed different tests within the same circuit. Five race walkers completed the long-distance race of 35 km, four race walkers completed the middle-distance race of 20 km, and finally, two race walkers completed the short-distance race of 10 km. The thermographic pattern of the hip was measured bilaterally, and skin temperature was measured in relation to the time and distance of the completion of the test.

### 2.2. Sample Characteristics

Eleven national and international walkers were recruited for this study. Of the eleven walkers, three belonged to the Spanish national walking team and one was a team world champion at the Oman 2022 World Championships. All the subjects had to meet the following inclusion criteria: (a) age 18–35 years; (b) BMI of 18.0–25.5 kg·m^2^; and (c) at least 3 years of training and competition experience in race walking at the national or international level. According to the TISEM protocol [19], subjects were excluded if they (a) had any cardiovascular pathology or were injured; (b) smoked or drank habitually or before the test; (c) took supplements or medication that could alter the thermoregulatory response before the test; (d) had received physiotherapy before the measurement. The pre-thermographic test diet was organized by the nutritionist according to the athletes’ sporting needs. The study was conducted in accordance with the Declaration of Helsinki for Research on Human Subjects [20] and was approved by the Ethics Committee of the Catholic University of Murcia (CE102102). All the participants were informed of the study procedures and signed informed consent forms.

### 2.3. Procedure

In the pre-competition familiarization session, the athletes underwent a medical examination (auscultation, blood pressure, standardized neurological tests, etc.) and a blood test to determine their health status. In addition, they received guidelines about the TISEM protocol [19] and were familiarized with the thermographic measurement protocol to be carried out before and after the 35 km, 20 km, and 10 km tests.

### 2.4. Tests Performed

#### 2.4.1. Thermography Protocol

**Figure 1:**
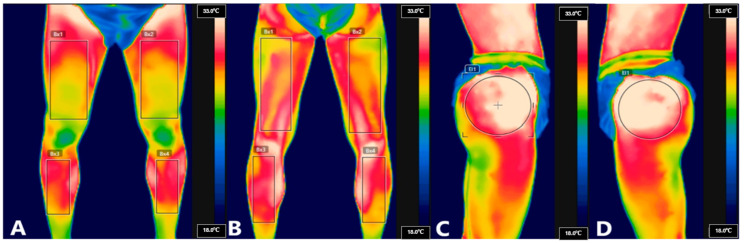
Thermographic Roi’s. (**A**) Anterior vision, (**B**) posterior vision, (**C**) right lateral vision, and (**D**) left lateral vision.

A Flir E75 model camera (Wilsonville, OR, USA) with an infrared resolution of 320 × 240 pixels and a thermal sensitivity of <0.04 °C was used to perform the thermographic test in this study. Measurement ranges were set between −20 and 120 °C. The emissivity was set at 0.98 in alignment with the bibliographic standards of other authors [21]. The athletes were positioned in shorts without shirts, leaving the hip joint free, on a 1.5 cm cotton pad in order to not generate temperature changes. The principal investigator started up the machine one hour before the first recording, which was logged at 7:15 a.m., and placed it on a tripod at a distance of one meter away from the sample measurement, at a point of 10° inclination. As described by other authors, in the first test, all the participants underwent a standard acclimatization period of 15–20 min in a room of 50 m^2^ at an average temperature of 22 °C (between 21 and 23), humidity of 42% ± 1%, and atmospheric pressure of 1 ATM. In this phase and after acclimatization, the first thermographic data were recorded at the pre-race basal level. In the second test (at the end of the competition), the athletes were instructed to go to the same room, which was kept at the same environmental conditions, in the shortest possible time, in order to perform the second thermographic recording. Across all the measurements, the regions of interest (ROIs) in the hip were defined using standardized bibliographic anatomical references [22]. Thermographic measurements were performed in various bipedal positions using the same model and specifications as previous similar studies [11]. To enhance accuracy and reliability in the analysis of anatomical regions, circular ROIs were selected for this study—unlike previous studies that only used quadrangular ROIs [11]—due to the nature of the joint being analyzed (hip). Quadrangular ROIs were applied for anterior and posterior views of the lower extremity (Figure 1). This approach allowed the anatomical boundaries of the structure to be respected in the thermographic images, improving the precision of the image analysis. Image processing was conducted by two blinded researchers using the Flir IR 6x research software following the analysis methods described in previous studies [23].

#### 2.4.2. Characteristics of the Race and External Environmental Conditions

The 35 km race of the Spanish Championship 2023 was held in Murcia in the city of Cieza (altitude: 187 m), latitude: 38°14′12″ north, longitude: 1°25′39″ west, on 26 February 2023. The race circuit was established in the center of the city on outdoor asphalt terrain without pendency, with an average ambient temperature during the test of 11.7 ± 1.5 °C, wind of 10 km/h from the west, humidity of 42%, and atmospheric pressure of 1 ATM as reported by the State Meteorological Agency AEMT [24] of the Autonomous Murcia Community. The test consisted of a road race on asphalt within a closed circuit and was approved by the RFEA, included in the National or Autonomous Calendar, and conducted in the presence of judges.

### 2.5. Tests Performed

The IBM Social Sciences software (SPSS, v.21.0, Chicago, IL, USA) was used for statistical analysis. The data are presented as mean ± SD. The homogeneity and normality of the data were checked with the Levene and Shapiro–Wilk tests, respectively. To analyze intragroup and intergroup differences, a three-way repeated measures ANOVA was performed for each ROI with a time factor (PRE vs. POST), distance factor (short vs. medium vs. long), and side factor (right (R) and left (L)). Tukey’s post hoc analysis was carried out if significance was found in the ANOVA models. Partial eta squared (η*p*^2^) was also calculated as the effect size for the interaction of all the variables in the ANOVA analysis. Partial eta square thresholds were used as follows: <0.01, irrelevant; ≥0.01, small; ≥0.059, moderate; ≥0.138, large. The significance level was set at *p* ≤ 0.05.

## 3. Results

**Figure 2:**
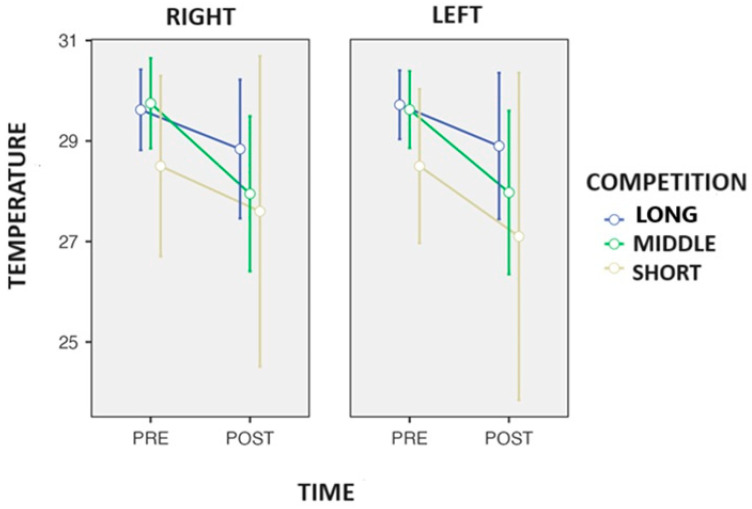
Changes in the surface skin temperature of the quadriceps after the end of the competition in the race walkers.

After the statistical analysis, a three-way repeated measures ANOVA found significant changes in Q temperature over time (*p* = 0.030: η*p*^2^ = 0.511) and Tukey’s post hoc observed a decrease in T° in the quadriceps (Figure 2) after the competition across all the distances (time) (−1.23 °C: *p* = 0.030). In addition, a trend was also observed in the side-to-competition interaction (*p* = 0.058: η*p*^2^ = 0.557).

**Figure 3:**
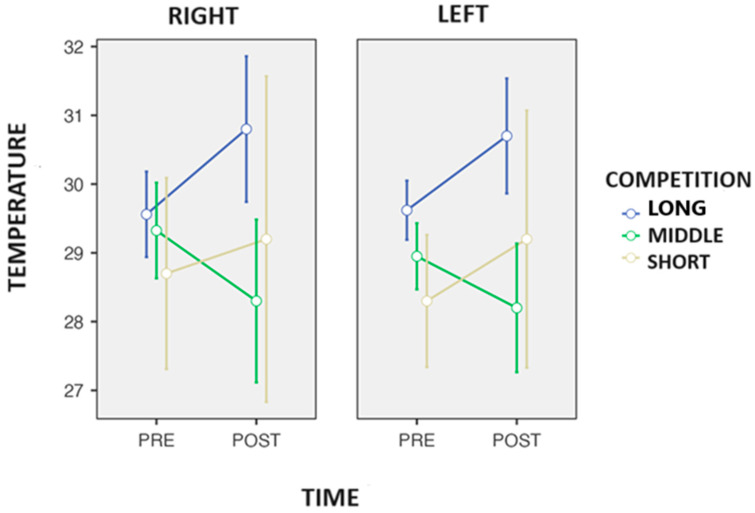
Changes in the surface skin temperature of the tibia after the end of the competition in the race walkers.

On the other hand, ANOVA detected a trend in the time-to-competition interaction (*p* = 0.057; η*p*^2^ = 0.558) in the tibia (Figure 3). Tukey’s post hoc showed that the middle-distance race walkers had −2.5 °C (*p* = 0.031) compared to the long-distance race walkers during the competition.

**Figure 4:**
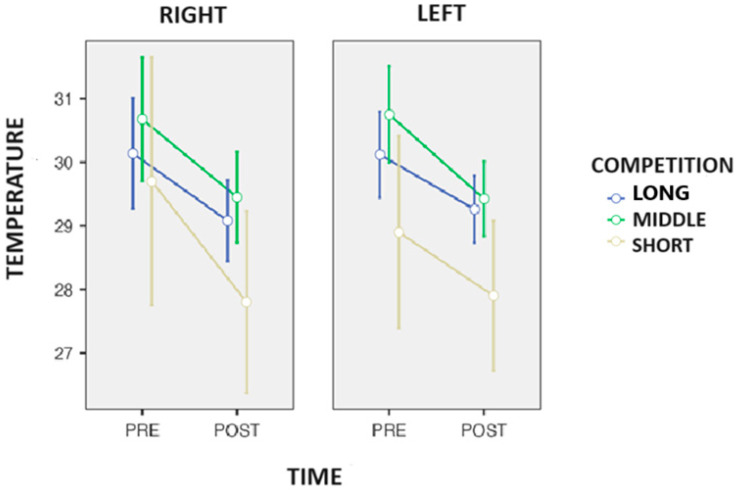
Changes in the surface skin temperature of the hamstrings after the end of the competition in the race walkers.

In relation to the H, ANOVA found a significant change over time (*p* = 0.002: η*p*^2^ = 0.765); furthermore, after performing Tukey’s post hoc, we observed a significant decrease in the hamstring (−1.23 °C; *p* = 0.002) after the end of the competition in the race walkers (Figure 4). In contrast, ANOVA only detected a trend in the time-to-side interaction (*p* = 0.083: η*p*^2^ = 0.369) in the hamstrings after the end of the competition. However, Tukey’s post hoc found a significant pre–post-competition decrease in the right H (−1.40 °C; *p* = 0.013) and left H (−1.10 °C; *p* = 0.006).

**Figure 5:**
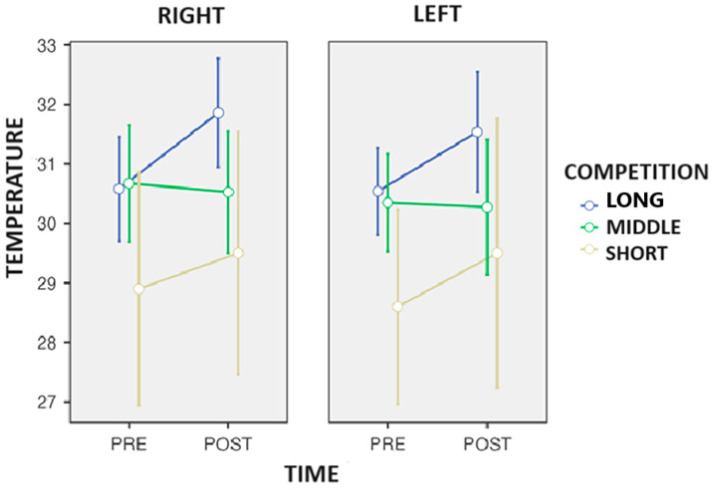
Changes in the surface skin temperature of the calves after the end of the competition in the race walkers.

Additionally, ANOVA detected a significant change in time in calves (*p* = 0.011; η*p*^2^ = 0.627), where Tukey’s post hoc showed a significant increase (time) (+0.50 °C; *p* = 0.011) in calves after the competition (Figure 5). In addition, ANOVA also found a significant change in the time-to-competition interaction (*p* = 0.010: η*p*^2^ = 0.730) in the calves; furthermore, Tukey’s post hoc found a significant increase in the long-distance competition (+1.14 °C; *p* = 0.011). ANOVA also detected a trend inside (*p* = 0.070; η*p*^2^ = 0.349), and after Tukey’s post hoc analysis, a trend was seen between the right and left sides (0.21 °C; *p* = 0.070) of the C after the end of the competition.

**Figure 6:**
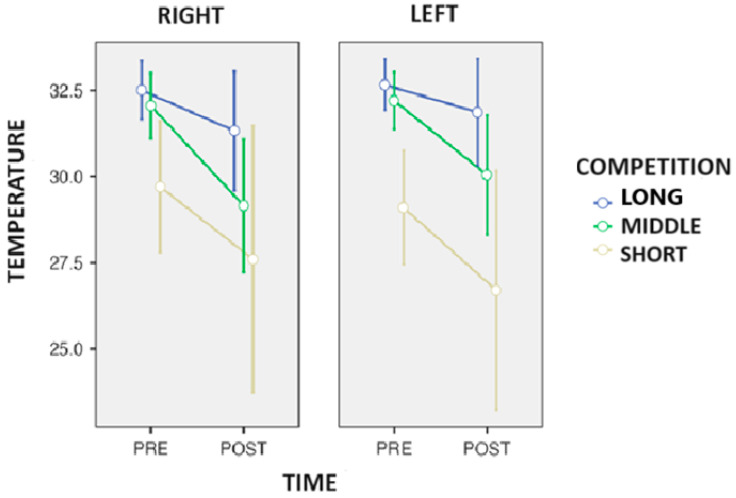
Changes in the surface skin temperature of the hip after the end of the competition in the race walkers.

Finally, ANOVA found a significant difference over time (*p* = 0.004: η*p*^2^ = 0.713) in the hip. In addition, Tukey’s post hoc showed a significant decrease in temperature (−1.92 °C; *p* = 0.004) in the hip after the competition (Figure 6).

## 4. Discussion

The main objective of this study was to evaluate metabolic stress using thermography in the lower limb after the Spanish Championship 2023 race walk. The main findings of this study revealed a decrease in skin temperature in all the analyzed anatomical regions, except for the back of the leg, after the test. Specifically, from the proximal to distal regions, a significant temperature reduction was observed in both the hip and quadriceps areas following the competition, regardless of the distance covered. The temperature decreased by −1.92 °C in the hip (*p* = 0.004) and by −1.23 °C in the anterior thigh region (*p* = 0.030).

In the tibial region, a temperature decrease was also observed, with the middle-distance walkers showing a more pronounced reduction of −2.5 °C (*p* = 0.031) compared to the long-distance walkers. In the posterior thigh region, a significant temperature decrease of −1.23 °C (*p* = 0.002) was noted after the competition. However, in contrast, a significant temperature increase of +0.50 °C (*p* = 0.011) was observed in the posterior leg region post-competition.

The analysis of metabolic response, pre-, during, and post-exercise by clinical thermography is a subject that is generating much debate due to, on the one hand, the ability of this technique to detect acute changes after physical exercise and, on the other hand, due to the difference in results obtained depending on the sport and the athlete [25,26]. In this regard, our results coincide with the findings shown by other authors in other sports, where a decrease in skin temperature has been observed after metabolic stress [27,28,29,30]. It should be noted that the comparison with another sport is because there are no previous studies with these characteristics. However, we believe that the physiological and metabolic processes can be extrapolated to this sample of athletes.

It has been well established by other authors that metabolic heat loss occurs through conduction, radiation, convection, and evaporation from a physical perspective [31]. In exercise physiology, blood flow adjusts to the metabolic demands of the musculoskeletal system, meaning that as a particular structure is stimulated, it requires vasodilation to enhance its oxygen supply. This results in reduced vascularization in less active regions, leading to a decrease in skin temperature, which can be observed thermographically. These findings are supported by our study, as although the posterior chain is generally more engaged than the anterior chain during race walking, the hip flexors play a crucial role during the propulsion and swing phases. They are essential for mitigating late-stage fatigue and, like the posterior muscles, undergo continuous activation and fatigue throughout the activity [32,33]. In addition, in previous studies, it has been observed that in the initial contact and swing phase, the gluteus maximus and biceps femoris have to be active to counteract the energy generated toward the ground and stabilize the lower limb joints at the time of loading, favoring the moment of translation of the trunk from posterior to anterior during the swing, and generating hip extension in knee extension [34]. The decrease in temperature observed at the anterior and posterior thigh level, and the thermal decrease at the hip level, could then be the result of this vascular change generated after metabolic stress. For this purpose, we believe that thermography could be of great help to coaches, athletic trainers, and physiotherapists to consider therapeutic and training strategies aimed at monitoring muscle response or delaying the onset of overload and consequential muscle fatigue at these levels, optimizing energy expenditure.

However, it should be noted that other authors have observed an increase in skin temperature after physical exercise [35,36,37]. These findings can be justified by the heating generated by the deeper cellular activation which is transferred to the more superficial anatomical areas. In fact, according to some authors, an increase in temperature after the onset of muscle fatigue in a given anatomical region may be associated with increased use or recruitment during competition compared to the basal condition of this anatomical region, which would generate tissue inflammation [36,38]. However, the relationship between muscle fatigue, inflammation, and temperature remains unclear. Some authors have observed that despite an increase in chemical markers in the blood directly related to muscle damage, a decrease in temperature, rather than an increase, has been noted [39].

In this regard, our results show an increase in temperature only at the level of the back of the leg, in the gastrocnemius region, something that had already been observed previously by other work carried out in race walkers. Specifically, these studies concluded that changes in skin temperature across different anatomical regions in endurance athletes are highly heterogeneous due to individual variations in athletes’ capabilities [11,40]. Consequently, determining a single energy expenditure pattern in these disciplines is challenging, as each athlete and category has its own anatomical and functional limitations [41]. However, considering that similar results were observed in this anatomical region across different environmental and running conditions, we could hypothesize that the triceps surae may have a predisposition to maintain higher metabolic activity compared to the other muscles in race walking. This hypothesis warrants further investigation in future studies. Therefore, we believe that the thermographic changes observed provide us with valuable information as they allow us to know what the thermographic pattern is like in short-, medium-, and long-distance race walkers, which, in turn, allows us to monitor the metabolic response in this type of athlete. Consequently, this information provides researchers and practitioners with the ability to carry out intervention protocols to prevent changes in temperature in the different anatomical areas involved in exercise.

Some authors have hypothesized that the varying results reported by different researchers may be influenced by factors such as exercise intensity, type of exercise, and duration. This variation in findings might reflect the continuous readjustment of thermoregulatory processes in humans based on different variables [42]. According to other authors, an initial increase in skin temperature is observed due to metabolic activation compared to the baseline condition. As the activity intensifies, however, a temperature decrease may occur, driven by vascular changes (convection) and the onset of sweating (evaporation). Once these responses take effect, the temperature decrease slows down until it stabilizes or fluctuates only slightly. At this point, if the physiological stress persists, micro tissue damage may occur, potentially causing a gradual temperature increase due to the inflammatory processes associated with this condition [11,23].

This model could explain the findings of our study, as half-marathon athletes exhibited the most significant thermal changes compared to the other athletes. It is possible that the long-distance group had already begun the process of overheating due to the prolonged duration of exercise, a condition that did not have sufficient time to develop in the middle-distance athletes, resulting in the significant thermal difference of −2.5 °C. However, to verify this model, it would be interesting to carry out other studies, where thermographic metabolic activity is monitored at various timepoints during exercise until maximum muscle fatigue is reached. This could substantially enhance our insights into whether the proposed models have a scientific basis or whether further research is needed to provide clear, grounded, and objective answers.

In summary, the findings of this study promote the use of thermography as a useful tool in the acute analysis of muscle functional activation and metabolic response in professional marching athletes. Although a priori, the small sample size could represent a limiting factor given it may alter the statistical analysis. However, it should be noted that this study has the results of professional athletes during the highest national competition and some of them reached the level of world champions, which is typically exclusive to a very small sample. Acknowledging the strengths and limitations of this study, future studies are required to better understand and clarify the relationship between the different physiological systems in this type of athlete. In this sense, thermography could represent a highly potent and valuable tool given its fast, inexpensive, non-invasive, practical, and portable nature. The fact that this technique enables obtaining metabolic and functional data sets before and immediately after a specific test in a short time could help trainers, physiotherapists, and nutritionists in planning protocols for the preparation and recovery of athletes in a more specific, ergonomic, and accurate way.

## 5. Conclusions

Our results confirmed that a change in skin temperature could be the result of a variation in acute metabolic and functional activation in the lower extremities of race walkers during competition, with infrared thermography being a tool capable of detecting such a change in a rapid and non-invasive manner. For this reason, its use for monitoring physiological responses during and after a competition may be justified.

## 6. Future Directions

Future research could evaluate the relationship between the blood markers of acute muscle fatigue and thermographic response in marching athletes in order to generate new knowledge for performance enhancement and the optimization of acute muscle response.

## Data Availability

Data generated or analyzed during this study are included in this article.
